# Supplementary material for: A transposable element insertion is associated with an alternative life history strategy
Source: Nat Commun. 2019 Dec 17;10:5757. doi: 10.1038/s41467-019-13596-2 (PMC6917731; doi:10.1038/s41467-019-13596-2)
Supplement: Supplementary file 1 — Supplementary Information [file 41467_2019_13596_MOESM1_ESM.pdf]

A transposable element insertion is associated with alternative life history strategies
Woronik et al. 2019

Supplementary Methods

DNA Extraction Protocols

Author Maria Celorio

Salting out reagents needed

Stock solutions

1M NaCl *(if 2M or 5M are available, they can be used instead)*
100 mM Tris-HCl pH 8.0
10 mM EDTA pH 8.0

Homogenising buffer

- 0.4M NaCl
- 10mM Tris-HCl pH8.0
- 2mM EDTA pH8.0

Directions:

1. Combine the stock solutions as necessary to reach the final concentrations.
2. Sterilize by autoclaving for 20 min at 15 psi (1.05 kg/cm2) on liquid cycle, then store the buffer at room temperature.

TE buffer

- 100 mM Tris-HCl (pH 8.0)
- 10 mM EDTA (pH 8.0)

Directions:

1. Combine the stock solutions as necessary to reach the final concentrations.
2. Sterilize by autoclaving for 20 min at 15 psi (1.05 kg/cm2) on liquid cycle, then store the buffer at room temperature.

10% SDS

- 10 g SDS (sodium dodecyl sulfate)
- 80 ml ddH_2O
- ** CAUTION ** SDS powder is hazardous. Prepare solution in fume hood.

Directions:

1. Dissolve 10g of SDS into 80 ml of ddH2O by stirring. Heat to 68 °C if necessary.
2. Add ddH2O until final volume is 100 ml.
3. Store at room temperature.

5M NaCl. Sterilize by autoclaving for 20 min

Proteinase K (20mg/ml). Suspend in ddH_2O.
Ethanol 95%, ice cold
Ethanol 70%
Agarose for running DNA gel
DNA ladder going up to 10Kb range, and down to around 500 bp.

Phenol chloroform extraction reagents needed

1. Phenol/chloroform/isoamyl alcohol (PCI) solution (25:24:1) DNase (RNase-and Protease-Free- Molecular Biology grade), pH 7.8-8.2
Important: acidic pH makes the DNA go into the phenolic layer while RNA goes into the aqueous layer
Suppliers:
http://www.sigmaaldrich.com/catalog/product/sigma/77617?lang=en&region=US
http://products.invitrogen.com/ivgn/product/15593031
http://www.fishersci.com/ecomm/servlet/fsproductdetail_10652_657663_29104_-1_0
2. Chloroform/isoamyl alcohol, 24:1 (Molecular Biology grade) *(make your own by mixing 96 ml chloroform and 4 ml isoamyl)*
Suppliers:
http://www.sigmaaldrich.com/catalog/product/fluka/25666?lang=en&region=US
https://new.fishersci.com/ecomm/servlet/fsproductdetail?aid=25168&&storeId=10652
3. Elution Buffer (10 mM Tris-HCl, pH 8.5) *(that is TE buffer)*
4. 100% Ethanol
5. 80% Ethanol

Salting out DNA extraction protocol

Protocol adapted by JA Hill from Green and Sambrook 2012 *Molecular Cloning vol. 1*.
October 6, 2014

A. Lysis
1. Add 800ul sterile homogenizing buffer to 2ml tube *(if 1.5ml are only available, first homogenize in 400 ul, then add 400ul after homogenization and mix by pipetting up and down)*
2. Add 200ul 10% SDS (2% final conc)
3. Add 25-50ul Proteinase K (20mg/ml) (depends how much tissue you have – approx. 50ul for 100mg)
4. Dissect out 50-100mg tissue, place in 2ml tube
5. Thoroughly grind tissue in buffer with pestle
6. Incubate 56°C 1 hour – overnight *(preferably overnight to increase yield)*

B. Either Salt extraction….
1. Add 4ul RNaseA (100mg/ml) to get rid of RNA
2. Mix gently, leave at RT for 5 minutes *(or 15 minutes if the samples are fairly fresh)*. Longer for very fresh or large amounts of tissue
3. Adjust sample to 0.2M NaCl using 5M stock, should be between 40ul and 50ul depending on proteinase K volume used *and mixed gently by inversion of tube. (here we have used 46 ul of 5M NaCl for each 50 ul of Proteinase K used).*
4. Centrifuge max speed 30mins *(here we have used 7500 rpm)*

******NOTE: To protect the integrity of genomic DNA clip the tips off the end of all pipette tips used to transfer solution containing gDNA*******

5. Transfer supernatant to two new 1.5ml tubes *(label in advance!)*. All cellular debris should be pelleted to the bottom of the tube allowing supernatant to be poured off into a new

tube then split into two parts of ~550ul each.

NOTE: After this step (no. 5 above for salt extraction), the protocol will normally follow the precipitation with ethanol (letter C. bellow), however we jump into the PacBio protocol and return to this step later on.

B. Or Phenol/Chloroform/Isoamyl Alcohol Extraction…

1. Start with 200 µL *(or 500 ul)* of material and a tube (label as TUBE 1). If necessary, bring the volume up to 200µL using the Elution Buffer ("EB") above.
2. Add an equal volume of the phenol/chloroform/isoamyl alcohol *(which should have pH 7.8 to 8.0)* solution to TUBE 1.
3. Vortex TUBE 1 vigorously for 1 minute.
4. Spin TUBE 1 solution at high speed for 5 minutes.
5. Remove ~180 µL *(if using 500ul initial vol. remove 375 ul)* of the top aqueous solution and place into a new tube, TUBE 2. Avoid picking up any of the phenol/chloroform/isoamyl alcohol phase.
6. Add 200 µL *(or 500ul if you started with 500ul in step 1.)* of EB to TUBE 1.
7. Vortex TUBE 1 vigorously for 1 minute.
8. Spin TUBE 1 solution at high speed for 5 minutes.
9. Remove as much of the top aqueous solution as possible from TUBE 1 *(about 375 ul if you started with 500 in step 1.)* without picking up any of the phenol/chloroform/isoamyl alcohol phase. Add the solution to TUBE 2.

(warning! Here probably we have two tubes number 2 due to the volumes, I made no comment but is very possible)

TUBE 2: Chloroform Back Extraction (the following steps are to be performed in TUBE 2)

10. Add equal volumes *(360-750 ul if you started with 500 ul in step 1., verify here)* of the chloroform/isoamyl *(I used only chloroform as indicated in the protocol page 46 in the book Molecular Cloning 1, Green & Sambrook)* alcohol solution to TUBE 2.
11. Vortex TUBE 2 vigorously for 1 minute.
12. Spin TUBE 2 solution at high speed for 5 minutes.
13. Remove as much of the top aqueous solution as possible *(about 500 ul if you started with 500 ul in step 1.)* and place into a new tube, TUBE 3. Avoid picking up any of the chloroform/isoamyl alcohol phase.

NOTE: The PacBio protocol moves then the ethanol precipitation (steps 14 to 28 in the original); however, we use the salt extraction – ethanol precipitation step instead, with no glycogen and no ammonium acetate.

C. Precipitation

1. Add 2 volume of ice-cold ethanol *(I used 99%)* (~1100ul).
2. Mix but inverting tube several times. White strands of gDNA should immediately appear.
3. Incubate tubes in ice-slurry for 30 minutes
4. Centrifuge 0°C max speed 15mins
5. Remove supernatant. A visible pellet of gDNA should remain in the tube.
6. Wash pellet by half filling the tube with 70% ethanol
7. Centrifuge 4°C max speed 3mins
8. Remove supernatant, pellets should remain.
9. Repeat steps 6-8 *(after repeat, spin briefly –in mini centrifuge- and take supernatant with 200ul pipette)*

10. Air-dry pellet on bench until fluid have evaporated, can be between 0.5-4 hours, but don't over dry or suspension will be difficult
11. Resuspend in 100ul TE buffer,

Software Versions and Parameters

Genome assembly: Raw reads were clone filtered using Stacks[1] (v.1.21, clone_filter), adaptors were trimmed (bbduk.sh ktrim=r k=23 mink=11 hdist=1), and low quality reads removed (bbduk2.sh ref= phix174_ill.ref.fa.gz k=27 hdist=1 qtrim=rl trimq=10 minlen=40 qout=33) using the BBmap software package (v. 34.86) (Bushnell B. sourceforge.net/projects/bbmap/). Cleaned reads were used as input for the AllPaths-LG (v. 50960, Haploidify=True, ploidy=2, targets=standard)[2] assembly pipeline. Metassembler (v. 1.5)[3] to merge our AllPathsLG and Falcon assemblies, using the AllPathsLG assembly as the primary assembly. The reference genome was annotated using MESPA (version 17_Aug_15)[4] with the primary protein set as the input (see transcriptome assembly for description of this protein set).

Bulk segregant analyses (BSA): Female Informative Cross: Raw reads were filtered and trimmed as described in the genome assembly section. Cleaned reads were mapped to the *C. crocea* reference genome using NextGenMap (v0.4.10, -i 0.09)[5]. SAMTOOLS (v1.2)[6] was used to filter (view -f 3 -q 20), sort, and index the bam files and generate mpileup files (mpileup –B) for the two pools and the Alba mother. Insertions and deletions were identified and masked using Popoolation2[7] and Popoolation[8], respectively (identify-indel-regions.pl --indel-window 5 and filter-sync-by-gtf.pl). Popoolation2[7] was used to convert the F1 mpileup files to a sync files (mpileup2sync.jar --min-qual 20) and calculate the allele frequency difference between Alba and orange pools (snp-frequency-diff.pl --min-count 3 --min-coverage 5). Male Informative Cross I: The same read cleaning, mapping and SNP calling pipeline used on the female informative cross was applied to this dataset. Male Informative Cross II: The same read cleaning, mapping and SNP calling pipeline used on the female informative and male informative I crosses was applied, except that there was no mother sequenced for the second male informative cross.

Genome wide association study: Raw reads were filtered and trimmed as descripted in the genome assembly section. Cleaned reads were mapped to the annotated reference genome using NextGenMap (v0.4.10, -i 0.6 -X 2000)[5]. Bam files were filtered and sorted using SAMTOOLS (v1.2, view -f 3 -q 20) [6]. A VCF file was generated using SAMTOOLS (v1.2, -t DP -t SP -Q 15)[6] and bcftools (v.1.2,-Ov –m -v) [6]. Read depth per site was calculated using VCFtools[9] (v0.1.13, --site-mean-depth). VCFtools was then used to call SNP sites with no more than 50% missing data, an average read depth between 15-50 across individuals, and a minimum SNP quality of 30 (--max-missing 0.5 --minQ 30 --remove-indels ----positions [file with sites that exhibited appropriate read depth]). An association analysis was performed with PLINK (v1.07, --assoc --adjust)[10] and a Benjamini & Hochberg step-up FDR control was applied.

Validating the Alba insertion: To validate that the contig carrying the Alba locus (*C. crocea* contig 12) was properly assembled we compared gene order across homologous regions in *Bombyx mori* (chromosome 15) and *Heliconius melpomene* (scaffold Hmel211009) by doing a tblastn search against Kaikobase v.3.2.2 (http://sgp.dna.affrc.go.jp/KAIKObase/, default settings) and blastp against LepBase (http://lepbase.org/), respectively, using protein sequences that were annotated to *C. crocea* contig 12 (Supplementary Fig 1A&B). Next, an

analysis of read depth using the 15 Alba and 15 orange re-sequeucing datasets mapped to our high-quality reference genome indicated that the locus was an Alba-specific insertion (Supplementary Fig 1C). Within this predicted insertion, MESPA (version 17_Aug_15)[4] annotated a *Jockey-like* transposable element (TE). To validate orange females lacked a TE insertion in this region we assembled the orange haplotye by performing a *de novo* genome assembly on the wild-caught, orange mother of male informative cross I using CLC Genomics Workbench v.5 (kmer size = 25, bubble size = 2000, https://www.qiagenbioinformatics.com/). MESPA (version 17_Aug_15)[4] was used to annotate the resulting genome assembly using the primary protein set (see transcriptome assembly for more about this protein set). We identified the orange contig carrying the *C. crocea BarH-1* homolog and aligned it with the Alba associated contig from our high quality reference genome using SLAGAN alignment via wgVISTA[11-14]. Regions of conservation between the two haplotypes were observed on both sides of the insertion, but not within, and neither MESPA nor a BLAST search could annotate a TE on the orange contig (Supplementary Fig 2). As a final bioinformatic validation we mapped the whole genome re-sequencing data to the orange assembly using SNAP[15] (-so -t 30 -F a -= -s 100 1000) (Supplementary Fig 2B&C). The expectation was that reads originating from the orange haplotype should map properly across the insertion site, while reads originating from the Alba haplotype would not due to the max 1000bp insert size set in SNAP. Reads from all orange individuals and some of the reads from 12 of the 15 Alba individuals, could properly map across the predicted insertion site on the orange haplotype. For Alba individuals with reads that could map across the insertion site, read depth within the insertion on the Alba haplotype indicates these individuals are likely heterozygous for Alba (Supplementary Fig 3) and the reads that can span the insertion site likely originate for the orange allele.

Transcriptome assembly, differential expression, and gene set enrichment analysis: Raw reads were adaptor filtered (bbduk2.sh ref=illumina_contaminents.fa, removeifeitherbad=t) and trimmed (reformat.sh qin=33 qout=33 requirebothbad=f verifypaired=t tossbrokenreads=t qtrim=t trimq=10) using the BBmap software package (v. 34.86) (Bushnell B. sourceforge.net/projects/bbmap/). Cleaned reads from all libraries were used in a *de novo* transcriptome assembly (Trinity version trinityrnaseq_r2013_08_14 with default parameters, kmer length = 25mers)[16]. To reduce the redundancy among contigs and produce a biologically valid transcript set, the tr2aacds pipeline from the EvidentialGene software package[17] was run on the raw Trinity assembly. The ‘okay primary’ sequence set was used as the reference transcriptome in all downstream analysis and called the primary set. The sixteen RNA-Seq libraries were mapped to the reference transcriptome using NextGenMap (v0.4.10, -i 0.09)[5]. SAMTOOLS (v1.2)[6] was then used to filter (view -f 3 -q 20), sort and index the sixteen bam files. SAMTOOLS (v1.2)[6] idxstats was then used to calculate the read counts per gene for each of the sorted bam files. These counts were then joined in a CSV file using csvjoin[18]. A differential expression analysis was conducted in R using EdgeR[19]. A Benjamini Hochberg correction was applied to the raw p values to correct for false discovery rate and differentially expressed genes were called (adjusted p value <0.05) (see Source Data). eggNOG-mapper (v.1)[20] was used with default settings to functionally annotate the transcriptome (Supplementary Data 5). The R package topGo[21] was used to conduct a gene set enrichment analysis on genes that exhibited > 1 or < -1 log fold change in the differential expression analysis (Supplementary Data 1-4).

References

1 Catchen, J., Hohenlohe, P. A., Bassham, S., Amores, A. & Cresko, W. A. Stacks: an analysis tool set for population genomics. *Mol Ecol* **22**, 3124-3140, (2013).

2 Gnerre, S. *et al.* High-quality draft assemblies of mammalian genomes from massively parallel sequence data. *Proc Natl Acad Sci U S A* **108**, 1513-1518, (2011).

3 Wences, A. H. & Schatz, M. C. Metassembler: merging and optimizing de novo genome assemblies. *Genome Biol* **16**, 207, (2015).

4 Neethiraj, R., Hornett, E. A., Hill, J. A. & Wheat, C. W. Investigating the genomic basis of discrete phenotypes using a Pool-Seq-only approach: New insights into the genetics underlying colour variation in diverse taxa. *Molecular Ecology* **26**, 4990-5002, (2017).

5 Sedlazeck, F. J., Rescheneder, P. & von Haeseler, A. NextGenMap: fast and accurate read mapping in highly polymorphic genomes. *Bioinformatics* **29**, 2790-2791, (2013).

6 Li, H. *et al.* The Sequence Alignment/Map format and SAMtools. *Bioinformatics* **25**, 2078-2079, (2009).

7 Kofler, R., Pandey, R. V. & Schlotterer, C. PoPoolation2: identifying differentiation between populations using sequencing of pooled DNA samples (Pool-Seq). *Bioinformatics* **27**, 3435-3436, (2011).

8 Kofler, R. *et al.* PoPoolation: a toolbox for population genetic analysis of next generation sequencing data from pooled individuals. *PLoS One* **6**, e15925, (2011).

9 Danecek, P. *et al.* The variant call format and VCFtools. *Bioinformatics* **27**, 2156-2158, (2011).

10 Purcell, S. *et al.* PLINK: a tool set for whole-genome association and population-based linkage analyses. *Am J Hum Genet* **81**, 559-575, (2007).

11 Couronne, O. *et al.* Strategies and tools for whole-genome alignments. *Genome Res* **13**, 73-80, (2003).

12 Brudno, M. *et al.* Glocal alignment: finding rearrangements during alignment. *Bioinformatics* **19**, i54-i62, (2003).

13 Mayor, C. *et al.* VISTA: visualizing global DNA sequence alignments of arbitrary length. *Bioinformatics* **16**, 1046-1047, (2000).

14 Frazer, K. A., Pachter, L., Poliakov, A., Rubin, E. M. & Dubchak, I. VISTA: computational tools for comparative genomics. *Nucleic Acids Research* **32**, W273-W279, (2004).

15 Zaharia, M. *et al.* Faster and More Accurate Sequence Alignment with SNAP. *arxiv*, doi:arXiv:1111.5572 (2011).

16 Grabherr, M. G. *et al.* Full-length transcriptome assembly from RNA-Seq data without a reference genome. *Nat Biotechnol* **29**, 644-652, (2011).

17 Gilbert, D. in *7th annual arthropod genomics symposium* (Notre Dame, 2013).

18 csvkit https://csvkit.readthedocs.io/en/latest/ (2016).

19 Robinson, M. D., McCarthy, D. J. & Smyth, G. K. edgeR: a Bioconductor package for differential expression analysis of digital gene expression data. *Bioinformatics* **26**, 139-140, (2010).

20 Huerta-Cepas, J. *et al.* Fast Genome-Wide Functional Annotation through Orthology Assignment by eggNOG-Mapper. *Mol Biol Evol* **34**, 2115-2122, (2017).

21 topGO: Enrichment Analysis for Gene Ontology v. R package version 2.34.0 (2018).

R code for differential expression analysis using EdgeR

```
#Using Bioconductor 3.8 (BiocInstaller 1.32.1), R 3.5.3 (2019-03-11).[Package edgeR version
3.24.3]
setwd("~/Desktop/nature_submission/nature_genetics/revise_for_nat_comm")
x <- read.delim("OKjoined_countscopy.tsv", sep = ',')

#Name columns and rows
head (x)
colnames(x) <- c("gene_name",
"Or_Ab_101","G","Or_Ab_103","G","Or_Ab_109","G","Or_Ab_111","G","Or_W_102","G","Or_W
_104","G","Or_W_110","G","Or_W_112","G","Al_Ab_113","G","Al_Ab_115","G","Al_Ab_105","G"
,"Al_Ab_107","G","Al_W_114","G","AL_W_116","G","Al_W_106","G","Al_W_108")
head(x)
rownames( x ) <- x[ , 1 ]

#Initial data visualization
groups_all_samples <- c(rep("ab", 8),rep("wing", 8))
all_samples <- x[,c(18,20,22,24,2,4,6,8,10,12,14,16,26,28,30,32)]
AS <- DGEList(counts=all_samples,group=groups_all_samples)
keep <- filterByExpr(AS)
AS <- AS[keep, , keep.lib.sizes=FALSE]
AS <- calcNormFactors(AS)
AS <- estimateDisp(AS)
plotMDS( AS , main = "MDS Plot for Count Data", labels = colnames( AS$counts ) )



##########################################################################
#Analyze Abdomen tissues in an Edge R matix

#Assign Groups
groupAb <- c(rep("Al_Ab", 4),rep("Or_Ab", 4))

#make abdomen only matrix
Ab <- x[,c(18,20,22,24,2,4,6,8)]

#create the edge R object
ABDGE <- DGEList(counts=Ab,group=groupAb)

#Filter lowly expressed genes out
keep <- filterByExpr(ABDGE)
ABDGE <- ABDGE[keep, , keep.lib.sizes=FALSE]

#Normalize
#normalizes for RNA composition by finding a set of scaling
#factors for the library sizes that minimize the log-fold changes between the samples for most
#genes. The default method for computing these scale factors uses a trimmed mean of Mvalues
(TMM) between each pair of samples [30].
#We call the product of the original library
#size and the scaling factor the effective library size. The effective library size replaces the
#original library size in all downsteam analyses.
```

```
ABDGE <- calcNormFactors(ABDGE)
ABDGE$samples
ABDGE <- estimateDisp(ABDGE)

#Redorder so results are in Alba context (ex. downregulated gene is downregulated in Alba)
ABDGE2<-ABDGE
ABDGE2$samples$group <- relevel(ABDGE2$samples$group, ref="Or_Ab")

#Conduct exact tests to determine DE genes
ab <- exactTest(ABDGE2)
topTags(ab)
dea <- decideTestsDGE(ab, p = 0.05)
summary(dea)

#Results
#Al_Ab-Or_Ab
#Down          17
#NotSig     12454
#Up            32
#negative logFC is down regulated in Alba

ab_sig_results<- as.data.frame(topTags(ab, n = 49))
ab_results<- as.data.frame(topTags(ab, n = 11148))

##########################################################################
#Analyze wing tissues in an Edge R matix

plotMDS(allwing , main = "MDS Plot for Count Data", labels = colnames( allwing$counts ) )
allwing<-x[,c(10,12,14,16,26,28,30,32)]
Wgroup <- c(rep("Or_W", 4) , rep("Al_W", 3))#assign groups
#Alba 108 is a major outlier so I remove it and use 3 Alba and 4 orange samples
W <- x[,c(10,12,14,16,26,28,30)]
WDGE <- DGEList(counts=W,group=Wgroup)
keep <- filterByExpr(WDGE)
WDGE <- WDGE[keep, , keep.lib.sizes=FALSE]

WDGE <- calcNormFactors(WDGE)
WDGE <- estimateDisp(WDGE)
WDGE2<-WDGE
WDGE2$samples$group <- relevel(WDGE2$samples$group, ref="Or_W")
wex <- exactTest(WDGE2)
topTags(wex)
wde <- decideTestsDGE(wex, p = 0.05)
summary(wde)

#Al_W-Or_W
#Down          9
#NotSig    11823
#Up           18
# -logFC is downregulated in Alba
```

```
w_sig_results<- as.data.frame(topTags(wex, n = 27))
w_results<- as.data.frame(topTags(wex, n = 11850))
```

R code for gene set enrichment analysis using topGO

```
# This is a  script to take in two files that have been pre processed
# 1) you need an annotation file that has a list of all genes tab then
# comma separated GO terms
# 2) you need a list of the candidate genes from that list you want to assess
# for being enriched compared to the genome.
#
# NOTE: these two files must have the following headers added to them.
# annotation=japonicus_aa.fasta.emapper.annotations
# candidate_file=japponicus_run6above95
# cut -f1,6 $annotation > $annotation.tsv
# echo 'Parent  GO_term' | cat - "$annotation".tsv > "$annotation".header.tsv
# echo 'geneid' | cat - "$candidate_file" > "$candidate_file".header.tsv
#
# the resulting *header.tsv files are then used in the script below
# 1st argument is the annotation file
# 2nd argument is the candidate set list
#
# note that the current setting for GO node size is 5, which
# is hard coded below for more robust results.
#
# example run
# Rscript GSEA_run_script.R gifuensis_aa.fasta.emapper.annotations.header.tsv
gifuensis_run6above95.header.tsv

# descriptions
# http://avrilomics.blogspot.com/2015/07/using-topgo-to-test-for-go-term.html

# new general run
rm(list=ls()) #clears all variables
objects() # clear all objects
graphics.off() #close all figures

library("topGO")
library("Rgraphviz")
library(openxlsx)

##########
# set variables
# making general script
# Rscript myscript.R batch.csv
# and invoke these in the myscript.R
args <- commandArgs(TRUE)
# dataset <- read.table(args[1],header=FALSE,sep=",",skip=1)
```

```
annotations=args[1]
candidate_list=args[2]
# here I will be only analyzing GO terms with at least 5 members,
# as this yield more stable results.
node_size=5

#### CORE BP #####
GO_category="BP"
geneID2GO <- readMappings(file = annotations)
geneUniverse <- names(geneID2GO)

genesOfInterest.bv <- read.table(candidate_list,header=TRUE)

genesOfInterest.bv <- as.character(genesOfInterest.bv$geneid)
geneList.bv <- factor(as.integer(geneUniverse %in% genesOfInterest.bv))
names(geneList.bv) <- geneUniverse

myGOdata.bv <- new("topGOdata", description="Candidate genes", ontology=GO_category,
allGenes=geneList.bv,  annot = annFUN.gene2GO, gene2GO = geneID2GO, nodeSize =
node_size)

# STATS#
# each GO term is tested independently, not taking the GO hierarchy into account
resultClassic <- runTest(myGOdata.bv, algorithm="classic", statistic="fisher")
# elim method processes the GO terms by traversing the GO hierarchy from bottom to top,
# ie. it first assesses the most specific (bottom-most) GO terms, and proceeds later
# to more general (higher) GO terms. When it assesses a higher (more general) GO term,
# it discards any genes that are annotated with significantly enriched descendant
# GO terms (considered significant using a pre-defined P-value threshold).
# This method does tend to miss some true positives at higher (more general)
# levels of the GO hierarchy.
resultElim <- runTest(myGOdata.bv, algorithm="elim", statistic="fisher")
# weight01 this is the default method used by TopGO, and is a mixture of the 'elim' and 'weight'
methods
resultTopgo <- runTest(myGOdata.bv, algorithm="weight01", statistic="fisher")
# when assessing a GO term, it takes into accoount the annotation of terms to the current term's
parents,
# and so reduces false positives due to the inheritance problem
resultParentchild <- runTest(myGOdata.bv, algorithm="parentchild", statistic="fisher")


# see how many results we get where weight01 gives a P-value <= 0.001:
mysummary <- summary(attributes(resultTopgo)$score <= 0.1)
numsignif <- as.integer(mysummary[[3]]) # how many terms is it true that P <= 0.001

allRes <- GenTable(myGOdata.bv,
                classicFisher = resultClassic,
                elimFisher = resultElim,
                topgoFisher = resultTopgo,
                parentchildFisher = resultParentchild,
```

```
                orderBy = "parentchildFisher", ranksOf = "classicFisher", topNodes = numsignif)


# write output
printGraph(myGOdata.bv, resultClassic, firstSigNodes = 5, fn.prefix = paste(candidate_list,
".",GO_category,".GSEA_graph_resultClassic", sep=""), useInfo = "all", pdfSW = TRUE)
printGraph(myGOdata.bv, resultTopgo, firstSigNodes = 5, fn.prefix = paste(candidate_list,
".",GO_category,".GSEA_graph_resultTopGo", sep=""), useInfo = "all", pdfSW = TRUE)
printGraph(myGOdata.bv, resultParentchild, firstSigNodes = 5, fn.prefix = paste(candidate_list,
".",GO_category,".GSEA_graph_resultParentchild", sep=""), useInfo = "all", pdfSW = TRUE)

write.table(allRes[,c(1,8)], file=paste(candidate_list,
".",GO_category,".GSEA_result.REVIGO.tsv", sep=""), sep = "\t", qmethod = "double", quote =
FALSE, row.names = FALSE, col.names = FALSE)
write.table(allRes, file=paste(candidate_list, ".",GO_category,".GSEA_result.tsv", sep=""), sep =
"\t", qmethod = "double", quote = FALSE, row.names = FALSE, col.names = TRUE)

write.xlsx(allRes, file = paste(candidate_list, ".",GO_category,".GSEA_result.xlsx", sep=""),
borders = "rows")

#### CORE MF #####
# new general run
objects() # clear all objects definded the previous run

##########
# set variables
# making general script
# Rscript myscript.R batch.csv
# and invoke these in the myscript.R
rm(myGOdata.bv,allRes,resultClassic,resultElim,resultTopgo,resultParentchild,GO_category)
#clears all variables
args <- commandArgs(TRUE)
# dataset <- read.table(args[1],header=FALSE,sep=",",skip=1)

annotations=args[1]
candidate_list=args[2]
# here I will be only analyzing GO terms with at least 5 members,
# as this yield more stable results.
node_size=5

#### CORE MF #####
GO_category="MF"
geneID2GO <- readMappings(file = annotations)
geneUniverse <- names(geneID2GO)

genesOfInterest.bv <- read.table(candidate_list,header=TRUE)

genesOfInterest.bv <- as.character(genesOfInterest.bv$geneid)
geneList.bv <- factor(as.integer(geneUniverse %in% genesOfInterest.bv))
names(geneList.bv) <- geneUniverse
```

```
myGOdata.bv <- new("topGOdata", description="Candidate genes", ontology=GO_category,
allGenes=geneList.bv,  annot = annFUN.gene2GO, gene2GO = geneID2GO, nodeSize =
node_size)

# STATS#
# each GO term is tested independently, not taking the GO hierarchy into account
resultClassic <- runTest(myGOdata.bv, algorithm="classic", statistic="fisher")
# elim method processes the GO terms by traversing the GO hierarchy from bottom to top,
# ie. it first assesses the most specific (bottom-most) GO terms, and proceeds later
# to more general (higher) GO terms. When it assesses a higher (more general) GO term,
# it discards any genes that are annotated with significantly enriched descendant
# GO terms (considered significant using a pre-defined P-value threshold).
# This method does tend to miss some true positives at higher (more general)
# levels of the GO hierarchy.
resultElim <- runTest(myGOdata.bv, algorithm="elim", statistic="fisher")
# weight01 this is the default method used by TopGO, and is a mixture of the 'elim' and 'weight'
methods
resultTopgo <- runTest(myGOdata.bv, algorithm="weight01", statistic="fisher")
# when assessing a GO term, it takes into accoount the annotation of terms to the current term's
parents,
# and so reduces false positives due to the inheritance problem
resultParentchild <- runTest(myGOdata.bv, algorithm="parentchild", statistic="fisher")


# see how many results we get where weight01 gives a P-value <= 0.001:
mysummary <- summary(attributes(resultTopgo)$score <= 0.1)
numsignif <- as.integer(mysummary[[3]]) # how many terms is it true that P <= 0.001

allRes <- GenTable(myGOdata.bv,
              classicFisher = resultClassic,
              elimFisher = resultElim,
              topgoFisher = resultTopgo,
              parentchildFisher = resultParentchild,
              orderBy = "parentchildFisher", ranksOf = "classicFisher", topNodes = numsignif)


# write output
printGraph(myGOdata.bv, resultClassic, firstSigNodes = 5, fn.prefix = paste(candidate_list,
".",GO_category,".GSEA_graph_resultClassic", sep=""), useInfo = "all", pdfSW = TRUE)
printGraph(myGOdata.bv, resultTopgo, firstSigNodes = 5, fn.prefix = paste(candidate_list,
".",GO_category,".GSEA_graph_resultTopGo", sep=""), useInfo = "all", pdfSW = TRUE)
printGraph(myGOdata.bv, resultParentchild, firstSigNodes = 5, fn.prefix = paste(candidate_list,
".",GO_category,".GSEA_graph_resultParentchild", sep=""), useInfo = "all", pdfSW = TRUE)

write.table(allRes[,c(1,8)], file=paste(candidate_list,
".",GO_category,".GSEA_result.REVIGO.tsv", sep=""), sep = "\t", qmethod = "double", quote =
FALSE, row.names = FALSE, col.names = FALSE)
write.table(allRes, file=paste(candidate_list, ".",GO_category,".GSEA_result.tsv", sep=""), sep =
"\t", qmethod = "double", quote = FALSE, row.names = FALSE, col.names = TRUE)
write.xlsx(allRes, file = paste(candidate_list, ".",GO_category,".GSEA_result.xlsx", sep=""),
borders = "rows")
```

Supplementary Figures

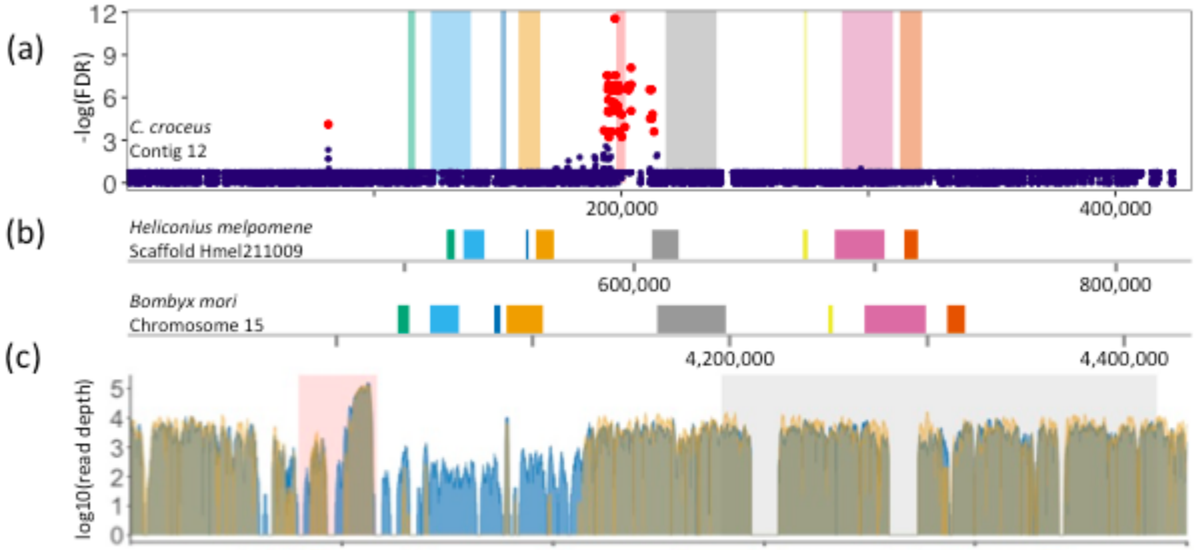

Supplementary Fig 1. Validating the transposable element insertion is unique to the Alba morph of *Colias crocea*. A) The location of Alba associated SNPs on the ~430 kb outlier contig identified in the GWAS. Red points are significant SNPs. Color bars indicate the location of genes along the contig. The red block contains the Jockey-like transposable element exons, gold block is the DEAD-box helicase and the grey block is BarH-1. Other color blocks represent other genes on the contig. B) Comparing gene order within the C. crocea Alba locus to gene order in Heliconius *melpomene* and *Bombyx mori*. In panel A and B the same color indicates the same gene. The conserved gene order among these species (shown in both panels A and B) indicates a well-assembled region, as synteny is highly conserved within Lepidoptera. Notably however both *H. melpomene* and *B. mori* both lack an annotated Jockey-like transposable element in this region (i.e the red color bar). C) Read depth differences within the Alba locus for an Alba female (blue plot) and an orange female (orange plot). Red and grey blocks contain the Jockey-like TE exons and BarH-1 respectively. Orange reads that map to Jockey exons presumably arise from copies of the TE located elsewhere in the genome. (See Supplementary Figure 2 for orange and Alba haplotypes). Gaps in read depth within *BarH-1* are repeat regions, however they are not morph specific.

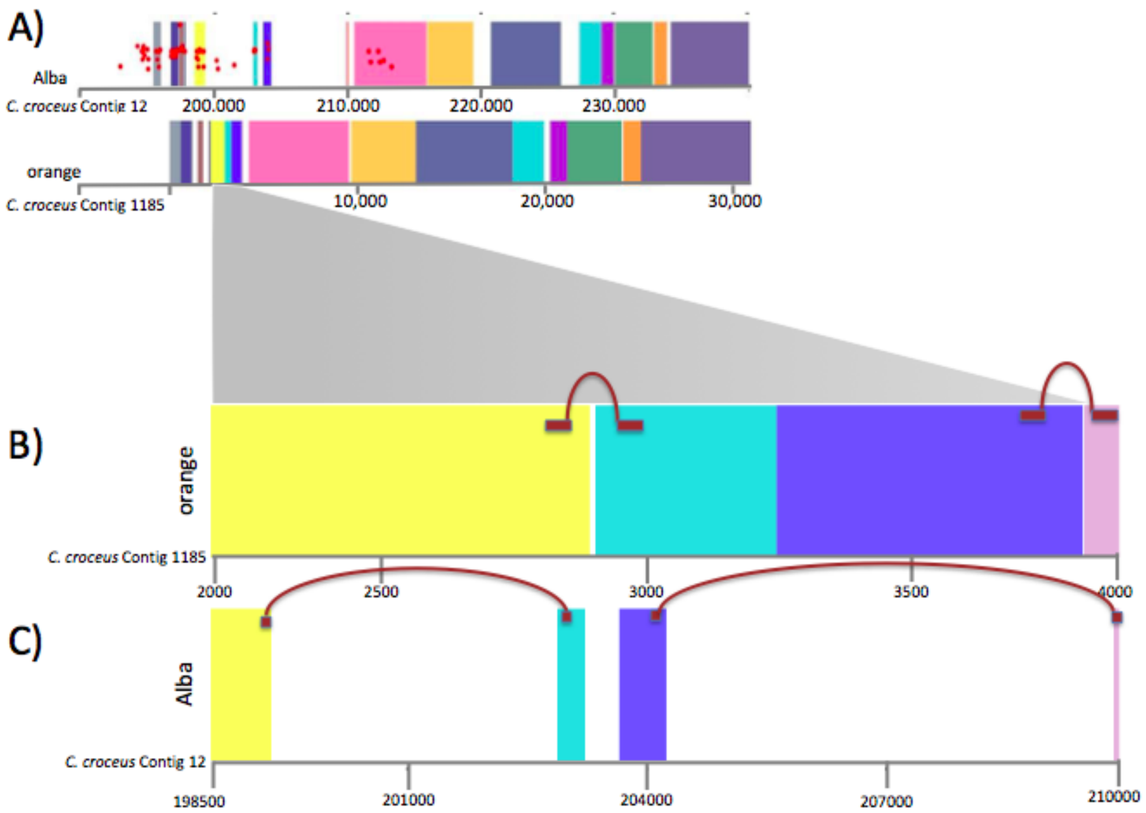

Supplementary Fig 2. An illustration of the technique used to validate the Alba specific insertions in the Alba haplotype via individual re-sequencing data. Using this haplotype alignment we identified two large insertions, ~ 5.6kb and ~ 3.6kb, that were present in the Alba haplotype but not the orange and contained TE sequence. A) Alignment of Alba and orange haplotypes. Color blocks indicate conserved regions. Red dots indicate the location of SNP sites that are significantly associated with Alba in the GWAS. B) A zoomed in representation of the orange haplotype, again color blocks indicate conserved regions. Red rectangles represent paired end reads used in the analysis because they maps to the regions of homology that flank the Alba specific insertion (i.e. span the insertion site). C) A zoomed in representation of the Alba haplotype. Color blocks are regions of conservation between haplotypes. Red blocks indicate how the same read pairs from panel B should map to the Alba haplotype. However because the insert size between the reads is above the threshold set during mapping the reads do not map.

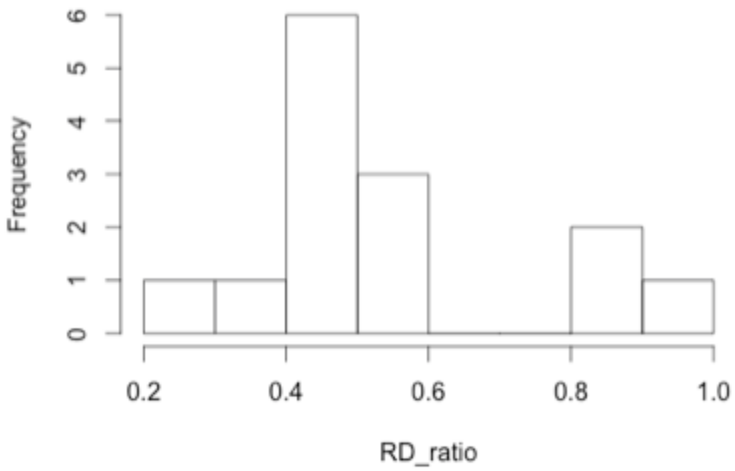

Supplementary Fig 3. A histogram to visualize the bimodal distribution in read depth (RD) difference ratios of Alba individuals. RD_ratio = read depth within the insertion (204244bp-209851bp)/read depth within a conserved genomic region (214000bp -218000bp). Individuals with a read depth ration >0.8 are presumable homozygous for Alba, while individuals with a read depth ratio < 0.6 are presumably heterozygous.

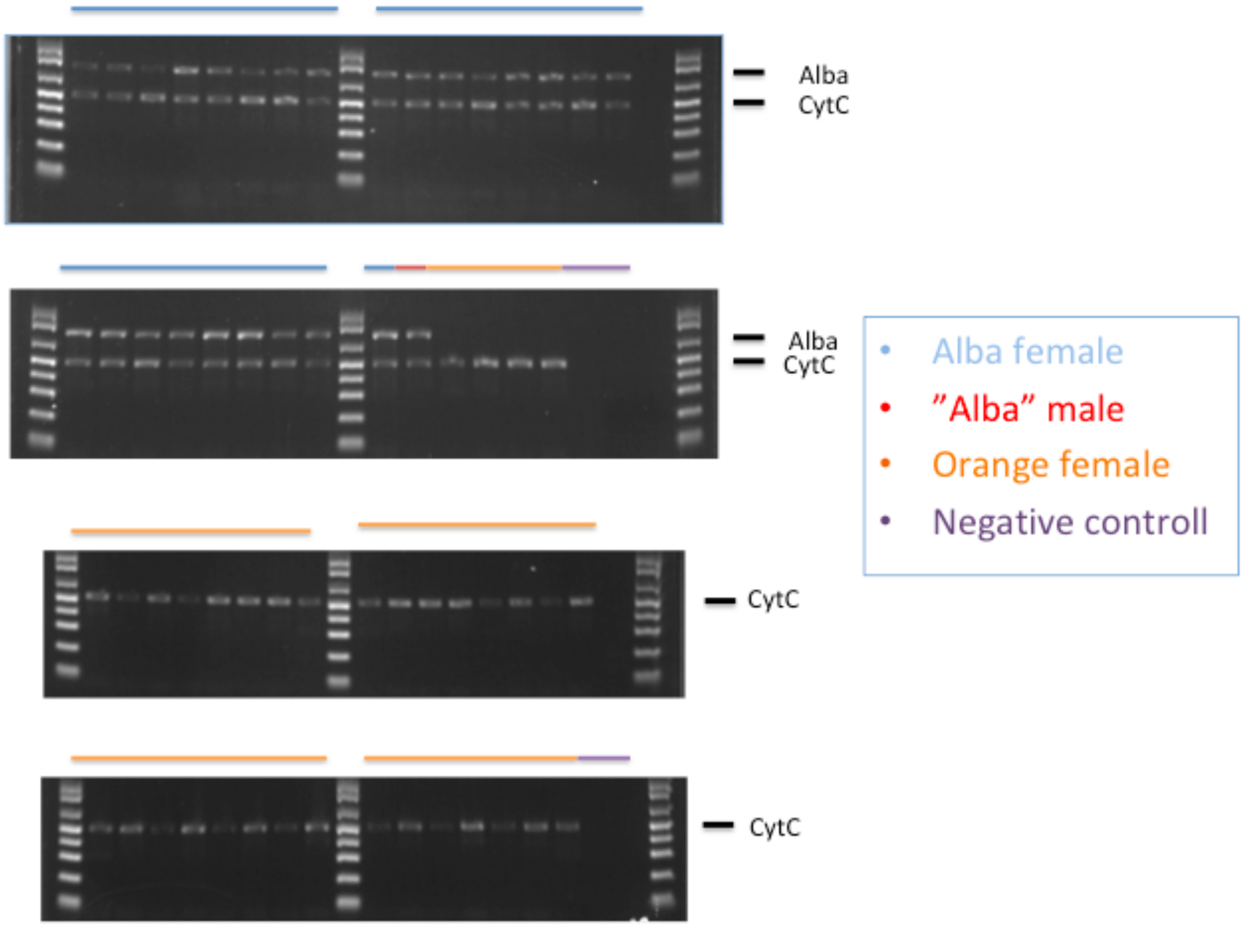

Supplementary Fig 4. PCR validation of the Alba insertion across wild caught individuals. Color bars along the top of the gel images indicate what sample is located within the well (see color key). *CytC* was used as a positive control in each reaction therefore Alba individuals should exhibit 2 bands (*CytC* and the Alba insertion), while orange should exhibit only one corresponding to *CytC*. Source data are provided as a Source Data file.

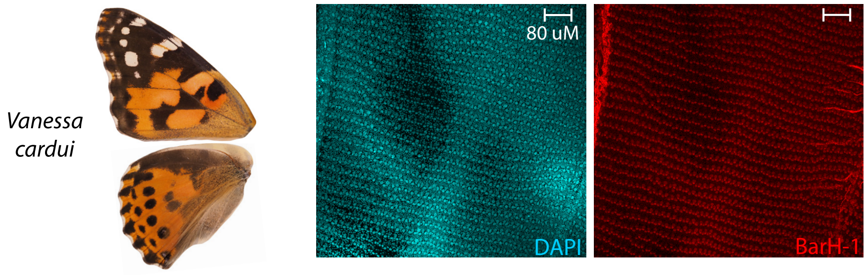

Supplementary Fig 5. BarH-1 antibody staining in *Vanessa cardui* pupal wings. From left to right: Adult *V. cardui* hind and forewings, DAPI (nuclei), and BarH-1 staining of developing *V. cardui* wings. Both the scale building and socket cells can be observed and express BarH-1. Scale bars are 80μM.

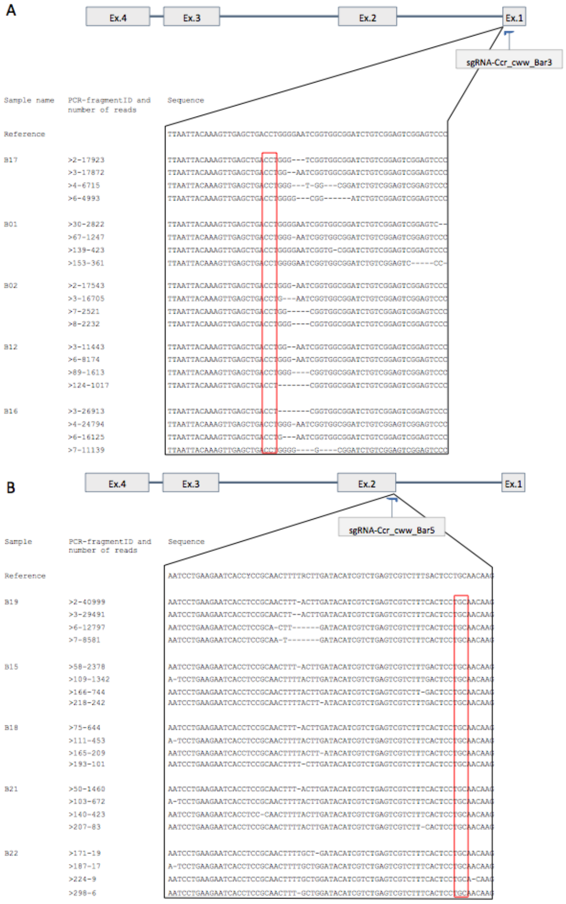

Supplementary Fig 6. Sequencing data to validate the CRISPR deletions. Sequence data from 5 samples injected with CcB3 (A) and 5 samples CcB5 (B). 4 of the most common PCR fragments containing deletions were selected, PCR-fragment ID (second column) is named by <rank>-<count> indicating that it also carried the allele.

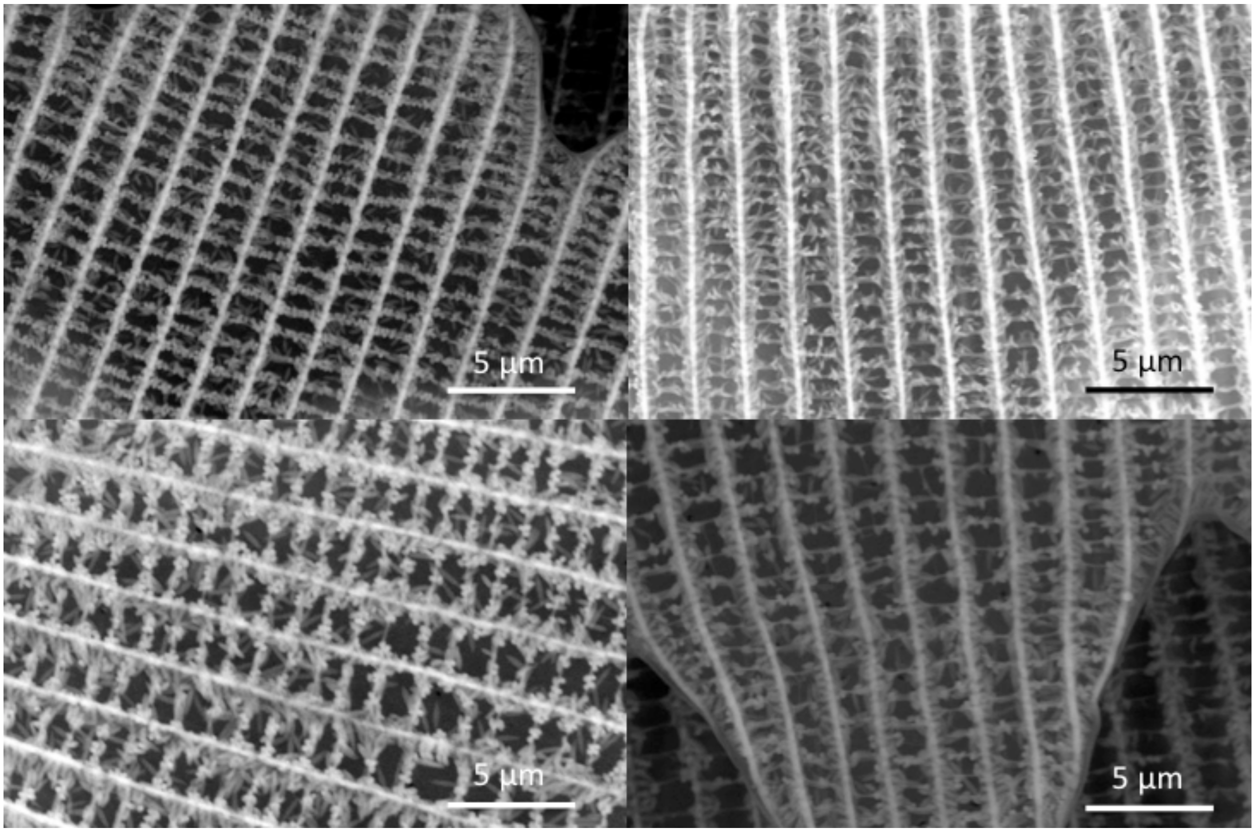

Supplementary Fig 7. Scanning electron microscope images illustrating the variation in pigment granule density that can occur between scale cells and individuals in *C. crocea* Alba females. We purposely searched for scales with the most pigment granules we could find. Scale bars are 5μM.

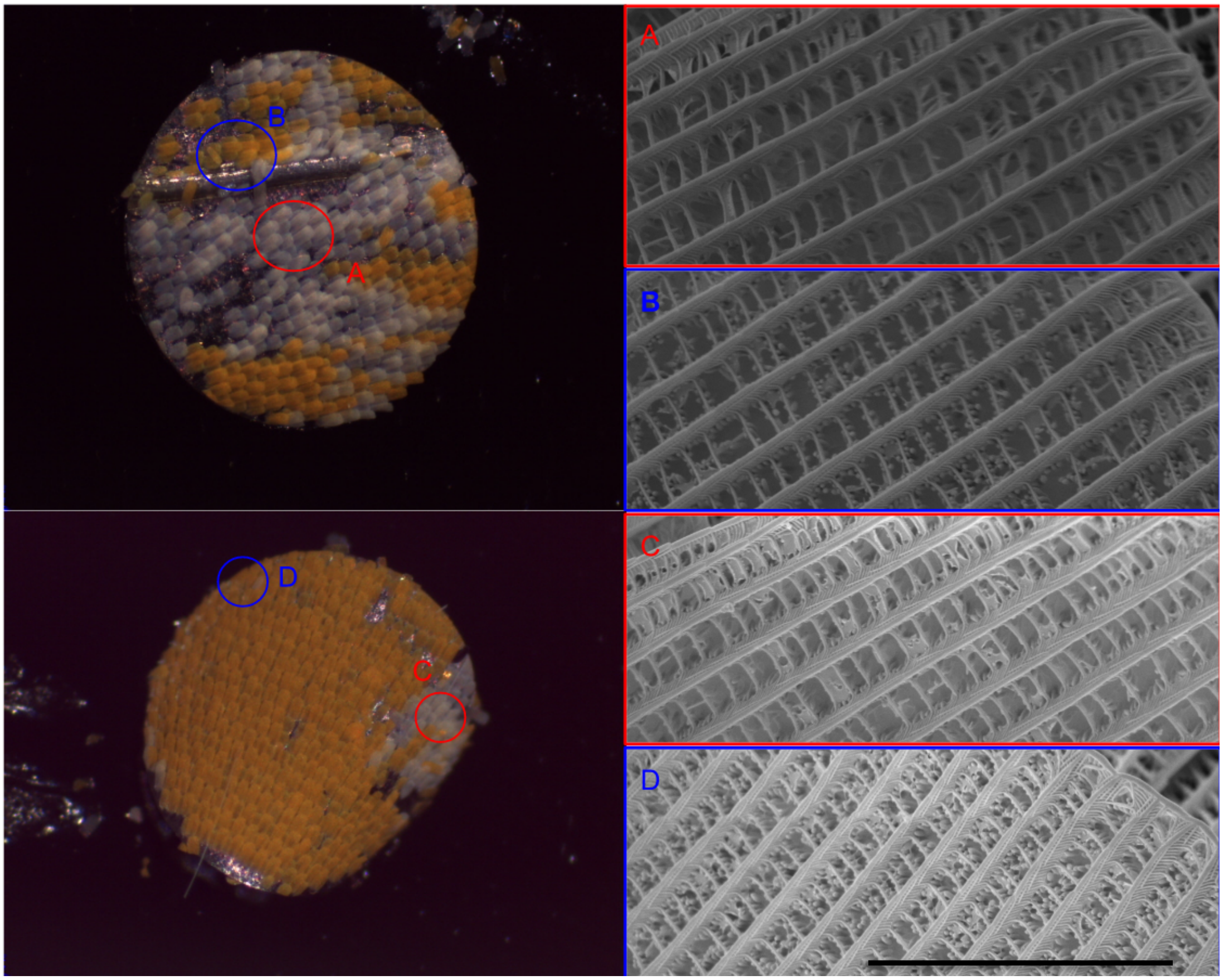

Supplementary Fig 8. Light microscope and scanning electron microscope images from CcB3-KO mosaic individual, upper row show mostly 1 white (A) and 1 orange (B) scale from a mostly white area, and lower row show 1 white (C) and 1 orange (D) from a mostly orange area. Scale bar is 10 μM.

Supplementary Tables

Supplementary Table 1. Percent of pupal development when color is first observed on pupal wings in *C. crocea*. Pupa were checked every 6 hours for pupation, first sign of color, and eclosion.

% of pupal development when color first appears on wing pad	SEX
50	F
78	M
74	M
74	M
74	M
74	F
74	M
58	M
75	F
90	M
73	F
75	F
69	M
63	M
90	M
72	M
51	M
72	F
91	M
88	M
72	F
73	F
94	M
65	F
91	F
60	F
77	M
53	F
73	M
73	F
86	F
76	M
75	F
83	M

75	M
84	M
62	M
76	M
76	F
64	M
76	M
64	F
62	F
95	M
65	M
64	M
64	M
62	M
64	M
64	F
65	M
59	F
84	F
64	M
76	M
66	F
67	F
74	F
68	F
65	F
77	F
74	M
75	F
80	F
56	M
70	M
75	M
66	F
75	F
69	F
71	F
66	M
64	M
75	F
70	F

73	M
62	M
75	F
74	M
75	M
75	F
73	F
60	F
69	F
68	M
66	M
64	F
60	M
55	F
64	F
82	F
75	F
59	F
75	M
69	M
62	F
52	M
75	F
94	F
75	M
41	M
72	M
54	F
76	M
72	F
61	F
61	F
64	F
61	M
55	F
49	F

Supplementary Table 2. Names of the contigs within the ~3.7 Mbp Alba locus identified via 3 rounds of bulk-segregant analysis.

Contig name
QPacBio.all_path_1
QPacBio.all_path_12
QPacBio.all_path_1371
QPacBio.all_path_1397
QPacBio.all_path_147
QPacBio.all_path_1586
QPacBio.all_path_1785
QPacBio.all_path_212
QPacBio.all_path_2235
QPacBio.all_path_2499
QPacBio.all_path_34
QPacBio.all_path_37
QPacBio.all_path_485
QPacBio.all_path_548
QPacBio.all_path_580
QPacBio.all_path_698
QPacBio.all_path_70
QPacBio.all_path_753
QPacBio.all_path_97

Supplementary Table 3. Name and sequence for the query region of gRNAs.

BarH-1 target sequences:	
**sgRNA-Ccr_cww_Bar1**	TCATGATCACGGATATCC
**sgRNA-Ccr_cww_Bar2**	AGAGACCTCAGCGCGCAC
**sgRNA-Ccr_cww_Bar3**	ATCCGCCACCGATTCCCC
**sgRNA-Ccr_cww_Bar4**	TGTATCAAGTAAAAGTTG
**sgRNA-Ccr_cww_Bar5**	TGAGTCGTCTTTCACTCC
**SgRNA-Cc_B6**	TGGCGGATCTGTCGGAGT
**SgRNA-Cc_B7**	TTCAGGATCGGATGGAGT

Supplementary Table 4. Observed phenotype in individuals with visible knockout by construct and genotype.

Sample	Sex	gRNA	Wing Color	Positive for Alba insertion	Eye phenotype	Ventral wing phenotype	Dorsal wing phenotype	Alba-PCR marker
B19	F	B3	Mosaic	Yes	Yes	Yes	Yes	Positive
B17	F	B5	Mosaic	Yes	Yes	Yes	Yes	Positive
B07	F	B1+2	OR	No	No	Yes	No	Negative
B15	F	B2	OR	No	Yes	Yes	No	Negative
B04	F	B3	OR	No	No	Yes	No	Negative
B06	F	B3	OR	No	Yes	Yes	No	Negative
B21	F	B3	OR	No	Yes	Yes	No	Negative
B22	F	B3	OR	No	Yes	Yes	No	Negative
B12	F	B3+4	OR	No	Yes	Yes	No	Negative
B13	F	B3+4	OR	No	Yes	No	No	Negative
B05	M	B3	OR	No	Yes	Yes	No	Negative
B20	M	B3	OR	No	Yes	No	No	Negative
B23	M	B3	OR	No	Yes	No	No	Negative
B03	M	B3+4	OR	No	Yes	No	No	Negative
B01	M	B5	OR	No	No	Yes	No	Negative
B18	M	B3	OR	Yes	Yes	No	No	Positive

Supplementary Table 5 Pigment granule counts for SEM images from wing scales from six wild-type *C. crocea* females.

Individual	Count per box			
	Box 1	Box 2	Box 3	Average
C. crocea orange original	101	124	114	113
C. crocea orange Cap 0045	179	165	164	169.3
C. crocea alba original	66	61	70	65.7
C. crocea alba 166	26	33	31	30
C. crocea alba 43 AW	126	113	76	105
C. crocea orange dark light	99	97	99	98.33333333

Supplementary Table 6. Pigment granule counts for SEM images from 10 wing scales from a single CRISPR/Cas9 *BarH-1* knockout female.

scale image	granules	color
37_Alba_mosaic_orange_sc1_close_110granules.jpg	110	orange
37_Alba_mosaic_ orange _sc2_close_001_119granules.jpg	119	orange
37_Alba_mosaic_ orange _sc3_close_99granules.jpg	99	orange
37_Alba_mosaic_ orange _sc4_close_86granules.jpg	86	orange
37_Alba_mosaic_ orange _sc5_close_119granules.jpg	119	orange
36_Alba_mosaic_white_sc1_close_001_31granules.jpg	31	white
36_Alba_mosaic_white_sc2_close_33granules.jpg	33	white
36_Alba_mosaic_white_sc3_close_23granules.jpg	23	white
36_Alba_mosaic_white_sc4_close_34granules.jpg	34	white
37_Alba_mosaic_white_sc1_close_40granules.jpg	40	white

Supplementary Table 7. Metadata for RNA Sequencing libraries

RNASeq_ID	Rearing ID	tissue	Color	% dev	260/280	conc bioanalyzer (ng/ul)
Or_Ab_101	55A	abdomen	orange	82	2.16	382.11
Or_W_102	55W	wing	orange	82	2.15	564.22
Or_Ab_103	126A	abdomen	orange	92	2.12	456.38
Or_W_104	126W	wing	orange	92	2.1	359.88
Or_Ab_109	134 A	abdomen	orange	90	2.03	632.83
Or_W_110	134W	wing	orange	90	2.07	354.96
Or_Ab_111	142A	abdomen	orange	88	2.13	308
Or_W_112	142W	wing	orange	88	2.14	750
Al_Ab_113	103A	abdomen	alba	88	2.15	513.89
Al_W_114	103W	wing	alba	88	2.15	702.73
Al_Ab_115	123A	abdomen	alba	92	2.01	48.97
Al_W_116	123W	wing	alba	92	2.11	337.95
Al_Ab_105	85A	abdomen	alba	88	2.08	221.72
Al_W_106	85W	wing	alba	88	2.06	313.33
Al_Ab_107	86A	abdomen	alba	88	2.09	298.34
Al_W_108	86W	wing	alba	88	1.96	183.43
