# Supplementary material for: A transposable element insertion is associated with an alternative life history strategy
Source: Nat Commun. 2019 Dec 17;10:5757. doi: 10.1038/s41467-019-13596-2 (PMC6917731; doi:10.1038/s41467-019-13596-2)
Supplement: Supplementary file 3 — Reporting Summary [file 41467_2019_13596_MOESM3_ESM.pdf]

nature research

Corresponding author(s): Alyssa Woronik
Christopher W Wheat

Last updated by author(s): Nov 8, 2019

Reporting Summary

Nature Research wishes to improve the reproducibility of the work that we publish. This form provides structure for consistency and transparency in reporting. For further information on Nature Research policies, see Authors & Referees and the Editorial Policy Checklist .

Statistics

For all statistical analyses, confirm that the following items are present in the figure legend, table legend, main text, or Methods section.

n/a	Confirmed	
☐	☒	The exact sample size (n) for each experimental group/condition, given as a discrete number and unit of measurement
☐	☒	A statement on whether measurements were taken from distinct samples or whether the same sample was measured repeatedly
☐	☒	The statistical test(s) used AND whether they are one- or two-sided<br>*Only common tests should be described solely by name; describe more complex techniques in the Methods section.*
☐	☒	A description of all covariates tested
☐	☒	A description of any assumptions or corrections, such as tests of normality and adjustment for multiple comparisons
☐	☒	A full description of the statistical parameters including central tendency (e.g. means) or other basic estimates (e.g. regression coefficient) AND variation (e.g. standard deviation) or associated estimates of uncertainty (e.g. confidence intervals)
☐	☒	For null hypothesis testing, the test statistic (e.g. F, t, r) with confidence intervals, effect sizes, degrees of freedom and P value noted<br>*Give P values as exact values whenever suitable.*
☒	☐	For Bayesian analysis, information on the choice of priors and Markov chain Monte Carlo settings
☒	☐	For hierarchical and complex designs, identification of the appropriate level for tests and full reporting of outcomes
☒	☐	Estimates of effect sizes (e.g. Cohen's d, Pearson's r), indicating how they were calculated

Our web collection on statistics for biologists contains articles on many of the points above.

Software and code

Policy information about availability of computer code

Data collection	Win-CATS 1.1.3.0 was used to collect the lipid data.
Data analysis	Genome assembly: Raw reads were clone filtered using Stacks1 (v.1.21, clone_filter), adaptors were trimmed (bbduk.sh ktrim=r k=23 mink=11 hdist=1), and low quality reads removed (bbduk2.sh ref= phix174_ill.ref.fa.gz k=27 hdist=1 qtrim=rl trimq=10 minlen=40 qout=33) using the BBmap software package (v. 34.86) (Bushnell B. sourceforge.net/projects/bbmap/). Cleaned reads were used as input for the AllPaths-LG (v. 50960, Haploidify=True, ploidy=2, targets=standard)2 assembly pipeline. Metassembler (v. 1.5)3 to merge our AllPathsLG and Falcon assemblies, using the AllPathsLG assembly as the primary assembly. The reference genome was annotated using MESPA (version 17_Aug_15)4 with the primary protein set as the input (see transcriptome assembly for description of this protein set).<br><br>Bulk segregant analyses (BSA): Female Informative Cross: Raw reads were filtered and trimmed as described in the genome assembly section. Cleaned reads were mapped to the C. crocea reference genome using NextGenMap (v0.4.10, -i 0.09)5. SAMTOOLS (v1.2)6 was used to filter (view -f 3 -q 20), sort, and index the bam files and generate mpileup files (mpileup –B) for the two pools and the Alba mother. Insertions and deletions were identified and masked using Popoolation27 and Popoolation8, respectively (identify-indel-regions.pl --indel-window 5 and filter-sync-by-gtf.pl). Popoolation27 was used to convert the F1 mpileup files to a sync files (mpileup2sync.jar --min-qual 20) and calculate the allele frequency difference between Alba and orange pools (snp-frequency-diff.pl --min-count 3 --min-coverage 5). Male Informative Cross I: The same read cleaning, mapping and SNP calling pipeline used on the female informative cross was applied to this dataset. Male Informative Cross II: The same read cleaning, mapping and SNP calling pipeline used on the female informative and male informative I crosses was applied, except that there was no mother sequenced for the second male informative cross.<br><br>Genome wide association study: Raw reads were filtered and trimmed as descripted in the genome assembly section. Cleaned reads were mapped to the annotated reference genome using NextGenMap (v0.4.10, -i 0.6 -X 2000)5. Bam files were filtered and sorted using SAMTOOLS (v1.2, view -f 3 -q 20) 6. A VCF file was generated using SAMTOOLS (v1.2, -t DP -t SP -Q 15)6 and bcftools (v.1.2,-Ov –m -v) 6. Read depth per site was calculated using VCFtools9 (v0.1.13, --site-mean-depth). VCFtools was then used to call SNP sites with no more than 50% missing data, an average read depth between 15-50 across individuals, and a minimum SNP quality of 30 (--max-missing 0.5 --

minQ 30 --remove-indels ----positions [file with sites that exhibited appropriate read depth]). An association analysis was performed with PLINK (v1.07, --assoc --adjust)10 and a Benjamini & Hochberg step-up FDR control was applied.

Validating the Alba insertion: To validate that the contig carrying the Alba locus (C. crocea contig 12) was properly assembled we compared gene order across homologous regions in Bombyx mori (chromosome 15) and Heliconius melpomene (scaffold Hmel211009) by doing a tblastn search against Kaikobase v.3.2.2 (http://sgp.dna.affrc.go.jp/KAIKObase/, default settings) and blastp against LepBase (http://lepbase.org/), respectively, using protein sequences that were annotated to C. crocea contig 12 (Supplementary Fig 1A&B). Next, an analysis of read depth using the 15 Alba and 15 orange re-sequeucing datasets mapped to our high-quality reference genome indicated that the locus was an Alba-specific insertion (Supplementary Fig 1C). Within this predicted insertion, MESPA (version 17_Aug_15)4 annotated a Jockey-like transposable element (TE). To validate orange females lacked a TE insertion in this region we assembled the orange haplotye by performing a de novo genome assembly on the wild-caught, orange mother of male informative cross I using CLC Genomics Workbench v.5 (kmer size = 25, bubble size = 2000, https://www.qiagenbioinformatics.com/). MESPA (version 17_Aug_15)4 was used to annotate the resulting genome assembly using the primary protein set (see transcriptome assembly for more about this protein set). We identified the orange contig carrying the C. crocea BarH-1 homolog and aligned it with the Alba associated contig from our high quality reference genome using SLAGAN alignment via wgVISTA11-14. Regions of conservation between the two haplotypes were observed on both sides of the insertion, but not within, and neither MESPA nor a BLAST search could annotate a TE on the orange contig (Supplementary Fig 2). As a final bioinformatic validation we mapped the whole genome re-sequencing data to the orange assembly using SNAP15 (-so -t 30 -F a -= -s 100 1000) (Supplementary Fig 2B&C). The expectation was that reads originating from the orange haplotype should map properly across the insertion site, while reads originating from the Alba haplotype would not due to the max 1000bp insert size set in SNAP. Reads from all orange individuals and some of the reads from 12 of the 15 Alba individuals, could properly map across the predicted insertion site on the orange haplotype. For Alba individuals with reads that could map across the insertion site, read depth within the insertion on the Alba haplotype indicates these individuals are likely heterozygous for Alba (Supplementary Fig 3) and the reads that can span the insertion site likely originate for the orange allele.

Transcriptome assembly, differential expression, and gene set enrichment analysis:
Raw reads were adaptor filtered (bbduk2.sh ref=illumina_contaminents.fa, removeifeitherbad=t) and trimmed (reformat.sh qin=33 qout=33 requirebothbad=f verifypaired=t tossbrokenreads=t qtrim=t trimq=10) using the BBmap software package (v. 34.86) (Bushnell B. sourceforge.net/projects/bbmap/). Cleaned reads from all libraries were used in a de novo transcriptome assembly (Trinity version trinityrnaseq_r2013_08_14 with default parameters, kmer length = 25mers)16. To reduce the redundancy among contigs and produce a biologically valid transcript set, the tr2aacds pipeline from the EvidentialGene software package17 was run on the raw Trinity assembly. The 'okay primary' sequence set was used as the reference transcriptome in all downstream analysis and called the primary set. The sixteen RNA-Seq libraries were mapped to the reference transcriptome using NextGenMap (v0.4.10, -i 0.09)5. SAMTOOLS (v1.2)6 was then used to filter (view -f 3 -q 20), sort and index the sixteen bam files. SAMTOOLS (v1.2)6 idxstats was then used to calculate the read counts per gene for each of the sorted bam files. These counts were then joined in a CSV file using csvjoin18. A differential expression analysis was conducted in R using EdgeR19. A Benjamini Hochberg correction was applied to the raw p values to correct for false discovery rate and differentially expressed genes were called (adjusted p value <0.05) (see Source Data). eggNOG-mapper (v.1)20 was used with default settings to functionally annotate the transcriptome (Supplementary Data 5). The R package topGo21 was used to conduct a gene set enrichment analysis on genes that exhibited > 1 or < -1 log fold change in the differential expression analysis (Supplementary Data 1-4).

The above text is available in the Supplementary Methods and the Code Availability section details where to find this information.

For manuscripts utilizing custom algorithms or software that are central to the research but not yet described in published literature, software must be made available to editors/reviewers. We strongly encourage code deposition in a community repository (e.g. GitHub). See the Nature Research guidelines for submitting code & software for further information.

Data

Policy information about availability of data

All manuscripts must include a data availability statement. This statement should provide the following information, where applicable:

- Accession codes, unique identifiers, or web links for publicly available datasets
- A list of figures that have associated raw data
- A description of any restrictions on data availability

Data availability: Raw reads, the reference genome and transcriptome can be accessed at NCBI Genbank nucleotide database using the following accession codes. Reference genome asembly: PRJNA588020, Female informative cross: PRJNA587696, Male informative cross I: PRJNA587518, Male informative cross II: PRJNA587716, 30 resequenced Alba and orange individuals: PRJNA587791, Transcriptome and RNA-Seq: PRJNA587755. Full SEM imgaes are available on FigShare [https://doi.org/10.6084/m9.figshare.10255664.v1].

Reviewer links to sequencing data below:

Bioproject PRJNA588020: de novo genome assembly
[https://dataview.ncbi.nlm.nih.gov/object/PRJNA588020?reviewer=oimft5a5fof477ehelpdh1mpjn]

Bioproject PRJNA587696: Female Informative Cross
[https://dataview.ncbi.nlm.nih.gov/object/PRJNA587696?reviewer=bv145o9gr8kmjqjh7biqoul22l]

Bioproject PRJNA587518: Male Informative Cross I
[https://dataview.ncbi.nlm.nih.gov/object/PRJNA587518?reviewer=jebnl5t272vjquj2i0kk38vdrc]

Bioproject PRJNA587716: Male Informative Cross II
[https://dataview.ncbi.nlm.nih.gov/object/PRJNA587716?reviewer=1i8ogufdov8dle77j7pq13h18p]

Bioproject PRJNA587791: 30 Alba and orange genomes for SNP association
[https://dataview.ncbi.nlm.nih.gov/object/PRJNA587791?reviewer=j7797rer129lglcd873uk12e3s]

Bioproject PRJNA587755: RNASeq and de novo transcriptome assembly
[https://dataview.ncbi.nlm.nih.gov/object/PRJNA587755?reviewer=9edht66223nnreibao3ifjg5d9]

Field-specific reporting

Please select the one below that is the best fit for your research. If you are not sure, read the appropriate sections before making your selection.

☐ Life sciences ☐ Behavioural & social sciences ☒ Ecological, evolutionary & environmental sciences

For a reference copy of the document with all sections, see nature.com/documents/nr-reporting-summary-flat.pdf

Ecological, evolutionary & environmental sciences study design

All studies must disclose on these points even when the disclosure is negative.

Study description	The focus of this study was to characterize the mechanism underlying an alternative life history strategy in Colias crocea butterflies. The alternative strategies are associated with a wing color polymorphism. We mapped the genetic basis of the wing color polymorphism using genomic data (3 round of bulk segregant analysis via pooled WGS and WGS of 30 individuals). Genomic findings were further investigated using antibody staining and CRISPR/Cas9 gene editing. The morphological basis of the color switch was investigated using scanning electron microscopy on wings. The color morph specific physiology was investigated using high performance thin layer chromatography and RNASeq.
Research sample	This study used wild caught butterflies from Catalonia Spain and Capri Italy. Butterflies from Catalonia were also captured and brought to Stockholm University where they were used to establish stocks for brood experiments. Colias crocea was selected because it exhibited the desired polymorphism and was a common species.
Sampling strategy	Sample sizes for sequencing were determined based on sample sizes successfully used in previous butterfly genomic studies that fine mapped traits of interest. These samples were also limited by sample availability. Genomic results were validated using antibody staining and CRISPR/Cas9 editing. RNASeq sample size was limited by the number of viable samples collected. 4 individuals of each morph and 2 types of tissue per individual. High performance thin layer chromatography was limited by brood size.
Data collection	Alyssa Woronik and Maria de la Paz Celorio-Mancera extracted nucleic acids. Library preparation and sequencing was conducted by the Beijing Genomics Institute (Shenzhen, China) or the Science for Life Laboratory (Stockholm, Sweden). Alyssa Woronik and Kalle Tunstrom dissected wings for antibody staining and Michael Perry conducted antibody staining and collected data using standard confocal microscopy. Alyssa Woronik, Michael Perry, Kalle Tunstrom, and Christopher Wheat conducted the CRISPR/Cas9 experiment. Kalle Tunstrom conducted CRISPR/Cas9 validation via sequencing. Alyssa Woronik collected samples for high performance thin layer chromatography and Reijo Käkelä and Philipp Lehmann conducted Folch extraction and high performance thin layer chromatography. Alyssa Woronik reared animals and conducted dissection for RNASeq work and library preparation and sequencing was conducted by the Science for Life Laboratory (Stockholm, Sweden). Alyssa Woronik and Kalle Tunstrom conducted scanning electron microscopy.<br><br>Butterflies from Spain were collected by Alyssa Woronik, Christopher Wheat, Constanti Stefanescu, Jason Hill, Lovisa Wennerström, Jofre Carnicer, and and Christina Hansen Wheat. Butterflies from Capri were captured by Oskar Brattstrom and Elishia Harji.
Timing and spatial scale	Field sampling was conducted from 2013-2017. Trips to field sites were made yearly at the beginning of the summer to collect wild animals to establish lab stocks needed for experiments. All butterflies in lab stocks originated from animals collected from Catalonia Spain.
Data exclusions	No data was excluded
Reproducibility	There were no attempts to repeat the experiment. However physiology experiments are congruent with previous published findings. Genomic findings were validated using antibody and CRISPR/Cas9 editing
Randomization	For genomic data individuals were grouped by morph. For 30 genomes used in association mapping individuals were wild caught from diverse population backgrounds (Capri, Italy and several field sites around Catalonia, Spain: Avall, Sant Antoni de Vilamajor and Aiguamolls de l'Empordà Nature Reserve). For lipid experiment larva were randomly assigned a temperature treatments.
Blinding	Blinding for genomic, CRISPR/Cas9, RNASeq, and antibody work was not possible. Lipid results were calculated by software.

Did the study involve field work? ☒ Yes ☐ No

Field work, collection and transport

Field conditions	Alfalfa meadows, April-September, Sunshine, >20oC
Location	Catalonia, Spain Alfalfa meadow. Sites Associated with the Catalan Butterfly Monitoring Scheme https://www.catalanbms.org/
Access and import/export	Sites Associated with the Catalan Butterfly Monitoring Scheme https://www.catalanbms.org/
Disturbance	No expected disturbance to sites. Researchers visited alfalfa meadows and captured butterflies on foot using nets.

Reporting for specific materials, systems and methods

We require information from authors about some types of materials, experimental systems and methods used in many studies. Here, indicate whether each material, system or method listed is relevant to your study. If you are not sure if a list item applies to your research, read the appropriate section before selecting a response.

Materials & experimental systems

n/a	Involved in the study	
☐	☒	Antibodies
☒	☐	Eukaryotic cell lines
☒	☐	Palaeontology
☐	☒	Animals and other organisms
☒	☐	Human research participants
☒	☐	Clinical data

Methods

n/a	Involved in the study	
☒	☐	ChIP-seq
☒	☐	Flow cytometry
☒	☐	MRI-based neuroimaging

Antibodies

Antibodies used	The Rabbit-anti-Bar antibody was used at 1:100, followed by secondary antibody staining with AlexaFluor-555-anti-Rabbit secondaries (Thermo Fisher Scientific, Waltham, MA, USA, Cat# A-31572) at 1:500 and counterstaining with DAPI at 1ug/uL in 1x PBS.
Validation	A Rabbit-anti-BarH-1 antibody was generated against the full length sequence of the Vanessa cardui BarH-1 homolog. Protein was generated by GenScript (Piscataway, NJ) and purified to >80% purity. DNA sequences to produce this protein were codon-optimized for bacterial expression and made via gene synthesis. GenScript injected resultant protein into host animals, collected serum for testing, and affinity purified the product using additional target protein bound to a column.

Animals and other organisms

Policy information about studies involving animals; ARRIVE guidelines recommended for reporting animal research

Laboratory animals	Offspring of wild caught butterflies were reared in facilities at Stockholm University.
Wild animals	C. croceus butterflies were collected from field sites associated with the Catalan Butterfly Monitoring Scheme. Primarily Alba and orange females were captured on foot using nets. Butterflies were then placed in butterfly envelopes and kept in a cooler. Individuals were fed sugar water daily. Butterflies either died of natural causes within flight cages at Stockholm University, or if their biological material was needed for DNA extraction they were frozen.
Field-collected samples	Female informative cross: A mated C. croceus Alba female was captured from a field site in Gava, Catalonia, Spain, in June 2013. This female was transported to Stockholm University where she oviposited on alfalfa (Medicago satvia). Her offspring were reared at Stockholm University in plastic cups during the summer of 2013 on alfalfa host plants at 27 ºC and a 16 hr day length. Upon pupation, individuals were moved to a 12 hr day length and 22 ºC. Emerged adults were frozen at -80 ºC.<br><br>Male informative cross I: A wild caught orange females oviposited on alfalfa and these offspring were reared on alfalfa in outdoor cages in Catalonia, Spain during September and October of 2014. Upon pupation, offspring were transported to Stockholm University where they eclosed in the lab. Upon eclosion individuals were stored at -80 ºC.<br><br>Male informative cross II: A male carrying Alba mated an orange female in the lab at Stockholm University. The mated female oviposited on alfalfa in the lab and offspring were reared in plastic cups on alfalfa at 27ºC with a 16hr daylength. Upon eclosion individuals were stored at -80 ºC.<br><br>Lipidomics experiment: Six Colias croceus Alba females were collected from field sites in Catalonia, Spain (Sant Antoni de Vilamajor and Aiguamolls de l'Empordà Nature Reserve) in July 2015, and subsequently oviposited on Medicago sativa (Linnaeus, Fabales:Fabaceae) in the laboratory at Stockholm University. Eggs were moved into individual plastic rearing cups covered with netting and randomly split between two temperature treatments (Hot: constant temperature of 27 °C, relative humidity ~54% for both larval and pupal development. Cold: constant temperature of 22 °C during larval development and a constant

temperature of 15 °C during pupal, relative humidity ~43%. A 16-hour day length was used in both treatments and both developmental stages). All individuals were visually checked at least every 12 h throughout the entirety of development. Hatch time from the egg, pupation, and eclosion were recorded (in hours). Larvae were reared on M. sativa collected from the area surrounding Stockholm University; it was provided ad libitum throughout development. Upon eclosion adults were stored at 4 °C until the next day. The next day the wet weights of the abdomen and thorax were recorded and the abdomen to thorax ratio calculated. Removed abdomens were stored at −80 °C for about one month before high performance thin layer chromatography (HPTLC) of lipids was performed.

RNASeq experiment: A mated C. croceus Alba female was captured at a field site in Catalonia, Spain, in April 2014. The wild caught female was transported to Stockholm University where she oviposited on alfalfa in the laboratory. These offspring were reared at Stockholm University during the summer of 2014 on alfalfa at 27 ºC and a 16 hr day length. When larvae reached the fifth instar they were checked at least every six hours and the pupation time of each individual was recorded. C. croceus pupa begin to produce pteridine wing pigments at ~ 70% of pupal development (Descimon, 1966); but, tissue was collected between 82% and 92% of pupal development (Supplement Table ST3) when the phenotype could be assigned with high confidence. The pupae were dissected in PBS solution, and then the abdomens and wings were flash frozen in liquid nitrogen and stored at -80 ºC.

Ethics oversight

In Sweden ethical approval is only required for experiments involving mammals, birds, reptiles, amphibians, fish and cyclostomes. Thus approval was not needed for this work and we did not seek it.

Note that full information on the approval of the study protocol must also be provided in the manuscript.
