# Supplementary material for: A transposable element insertion is associated with an alternative life history strategy
Source: Nat Commun. 2019 Dec 17;10:5757. doi: 10.1038/s41467-019-13596-2 (PMC6917731; doi:10.1038/s41467-019-13596-2)
Supplement: Supplementary file 4 — Description of Additional Supplementary Files [file 41467_2019_13596_MOESM4_ESM.pdf]

Description of Additional Supplementary Files

File Name: Supplementary Data 1
Description: Gene set enrichment analysis results from topGO for genes upregulated in Alba abdomens

File Name: Supplementary Data 2
Description: Gene set enrichment analysis results from topGO for genes downregulated in Alba abdomens

File Name: Supplementary Data 3
Description: Gene set enrichment analysis results from topGO for genes downregulated in Alba wings

File Name: Supplementary Data 4
Description: Gene set enrichment analysis results from topGO for genes upregulated in Alba wings

File Name: Supplementary Data 5
Description: Transcriptome GO term annotation results from EggNOG
